# Lessons to be learned when designing comprehensible patient‐oriented online information about temporomandibular disorders

**DOI:** 10.1111/joor.13798

**Published:** 2024-07-21

**Authors:** Hande Uzunçıbuk, Maria Maddalena Marrapodi, Vincenzo Ronsivalle, Marco Cicciù, Giuseppe Minervini

**Affiliations:** ^1^ Department of Orthodontics, Dentistry Faculty Trakya University Edirne Turkey; ^2^ Department of Woman, Child and General and Specialist Surgery University of Campania “Luigi Vanvitelli” Naples Italy; ^3^ Department of General Surgery and Medical‐Surgical Specialties, School of Dentistry University of Catania Catania Italy; ^4^ Saveetha Dental College and Hospitals, Saveetha Institute of Medical and Technical Sciences (SIMATS) Saveetha University Chennai India; ^5^ Multidisciplinary Department of Medical‐Surgical and Dental Specialties University of Campania “Luigi Vanvitelli” Naples Italy

**Keywords:** gunning fog index, orthodontics, readability, temporomandibular disorders, websites

## Abstract

**Background:**

Temporomandibular disorders (TMD) are a prevalent ailment with a global impact, affecting a substantial number of individuals. While some individuals are receiving treatment from orthodontists for TMD, a significant proportion of individuals obtain knowledge through websites.

**Objectives:**

Our purpose had been to evaluate, from a patient‐oriented perspective, the readability of home pages of websites scored in the 10 most prominent devoted to TMD. We also determined what level of education would have been needed to get an overview of the information on the websites under scrutiny. This approach ensures that our findings are centred on the patient experience, providing insights into how accessible and understandable websites about TMD.

**Methods:**

We determined the top 10 patient‐focused English language websites by searching for ‘temporomandibular disorders’ in the ‘no country redirect’ plugin of the Google Chrome browser (www.google.com/ncr). The readability of the texts was assessed using the Gunning fog index (GFI), Coleman Liau index (CLI), Automated readability index (ARI) Simple Measure of Gobbledygook (SMOG), Flesch Kincald grade level (FKGL), Flesh reasing ease (FRE) (https://readabilityformulas.com).

**Results:**

The mean Flesch reading ease index score was determined to be 48.67, accompanied by a standard deviation of 15.04 and these websites require an average of 13.49 years of formal education (GFI), with a standard deviation of 2.62, for ease of understanding.

**Conclusion:**

Our research indicates that a significant proportion of websites related to TMD can be defined as a level of complexity that exceeds the ability to read comprehension of the general population.

## Graphical abstract

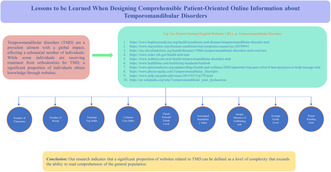

## 1 INTRODUCTION

Temporomandibular disorders (TMD) is a comprehensive term that encompasses both discomfort and dysfunction of the mastication muscles as well as the temporomandibular joints, which serve as the connection between the mandible and the cranial base. The primary characteristic of significance is discomfort, often presenting as pain, which is the most common reason individuals seek help from general dental practitioners or specialists.^1^ The pain can range from mild discomfort to very challenging and disabling pain, which significantly interferes with daily activities.^2^ It is usually accompanied by limited mandibular mobility and auditory signs arising in the TMJ during jaw movement, for example, clicking, popping or grinding.^3^ There are even more complications from TMD chronic pain, such as headaches, pain in the neck area and even alteration of posture after some time because of the persisting discomfort. This is usually a reason that drives most patients to seek assistance since it interferes with quality of life maximally. Though TMD does not pose a life danger, it impacts the quality of an individual's life.^4^ The aetiology of TMD exhibits a range of factors, with notable contributors encompassing psychological stress, bruxism, traumatic incidents, rheumatoid arthritis and muscular spasms.^5^

Orthodontics is that branch of dentistry concerned with the straightening and correcting teeth and jaws. The relation between TMD and orthodontics has been a matter of controversy. Several studies have suggested that certain occlusal disturbances and malocclusions increase the risk for TMD symptoms.^6^, ^7^, ^8^, ^9^, ^10^, ^11^ Malocclusion is a condition characterised by misalignment of teeth and jaws, which prevents their proper closure.^12^ Some types of malocclusion, mainly extensive overbite or crossbite, can contribute because they will put pressure on and stress the TMJ and adjoining tissues. Several medical literature have explained this anatomy to lead toward TMD symptoms. Research has been conducted to estimate the interference of malocclusion in TMD symptomatology, showing that some kinds of maloclosures may increase the risk for TMD. These are big overjet, anterior open bite and unilateral crossbite.^13^, ^14^, ^15^ The misplacement of teeth can result in the generation of imbalanced stresses on the temporomandibular joint, hence elevating the susceptibility to TMD. There are also two kinds of bruxism: sleeping and daytime. Sleep bruxism mostly happens in the process of sleeping and is not conscious; it is generally related to different types of stress, sleeping disorders or even some kind of medicine. In contrast, daytime bruxism happens typically in the awake state and could be a response to stress, anxiety or concentration. Both forms of bruxism can cause significant wear on the teeth and strain on the temporomandibular joint, but their triggers and patterns can differ. An improper bite can lead to muscle strain and increase the likelihood of bruxism, which in turn can inflict harm upon the musculature and structural integrity of the jaw.^6^, ^16^, ^17^, ^18^

The treatment of TMD may exhibit variability contingent upon the intensity and aetiology of the associated symptoms. Pain medications, hot and cold therapy, dental night guards, and physical therapy are employed in the treatment of TMD, alongside orthodontic interventions, to mitigate and rectify associated symptoms.^19^, ^20^

Although several studies are pointing to a possible relationship between orthodontic treatment and TMD, it is still true that the literature indicates this complex relationship must not yet be understood.^7^, ^8^, ^21^ The circumstances of each individual are distinct, necessitating personalised treatment that addresses their distinctive requirements. However, patients suffering from TMD frequently resort to websites as a means of acquiring knowledge pertaining to their medical issue.^22^, ^23^, ^24^, ^25^, ^26^, ^27^ There is an increasing level of public awareness of health‐related concerns, leading patients to independently acquire over‐the‐counter devices or different treatment protocols through websites for the purpose of managing conditions such as pain or bruxism. This trend is observed without the involvement of dental or medical professionals in the decision‐making process.^22^, ^28^

Nevertheless, it is important to point out that the reliability of the information accessible on the websites may be unclear at sometimes. The diminished comprehensibility of digital content and the constrained medical literacy within the broader populace might hinder patients' complete comprehension of health‐related websites.^29^, ^30^ A website's readability depends on several factors. These are content, vocabulary, sentence structure, display, and typography. When most people mention the term ‘Typography,’ they consider all factors associated with type. This refers to the size of the font, height of the line, and line length. ‘Readability and Comprehension’ means how easily the reader can read and comprehend a text from a website. The objective of this study was to evaluate the readability of the top 10 websites focused on patient‐oriented information on TMD. Moreover, the educational level required to understand the information that is presented on the investigated websites was to be established. Through the examination of the home pages of these websites, the researchers aimed to ascertain if the content supplied exhibited a level of accessibility suitable for the general readership or necessitated a higher degree of educational attainment for comprehension.

## 2 MATERIALS AND METHODS

The researchers utilised the Google Chrome browser, equipped with the ‘no country redirect’ extension, to conduct a search for the term ‘temporomandibular disorders’.

The study was performed between January and August 2023. The objective was to identify the initial 10 patient‐oriented websites in the English language. The selection of these websites was conducted by two independent researchers (H.U. and M.M.M.) who ensured that the chosen websites were those that appeared most prominently in the search results. These websites, filtered by their ranking on the search engine results page (SERP), deal with TMD from the patient's point of view to guarantee consistency and relevance.

Inclusion criteria for the selection of websites included:

1. The website must be written in English
2. The website must provide patient‐oriented information on TMD
3. The website must have a minimum of 500 words on the home page

Websites were excluded if they were non‐English, aimed at healthcare professionals rather than patients, or contained less than 500 words. The textual content from the identified websites was then extracted by copying and pasting into Microsoft Word. The material was modified with the purpose of eliminating errors and unnecessary details that may impede the study.

In order to evaluate the comprehensibility of the content in the website, the researchers employed six widely accepted readability assessments:

1. Gunning fox index (GFI): GFI is a metric employed for evaluating the level of complexity in an article. The method calculates the duration of education necessary for comprehending a text by considering the mean sentence length and the proportion of advanced terminology. GFI is used due to its basis on the assumption that the number of years of formal education necessary to comprehend a text at initial reading is predictive of its readability. As a result, the findings suggest that the level of formal education necessary for individuals to comprehend the text effortlessly on their first reading.^31^
2. Coleman Liaeu index (CLI): CLI is a formula used to measure the readability of a text by considering the average number of characters in 100 words and the average sentence length.^32^, ^33^ In contrast to previous assessments of readability, the CLI does not take into account the syllable count of a given text.^33^ The CLI, in addition to other readability assessments such as the GFI and the simple measure of gobbledygook (SMOG) readability formula, yields a grade level result, where lower values correspond to higher levels of readability.^34^, ^35^, ^36^
3. Automated readability index (ARI): ARI is a computer software or program specially developed to assess readability in any text. This is done on the basis of two critical parameters: an average number of letters per word and an average of words per sentence. The higher the score in this index, more challenging and complex it will be concerning its readability. The ARI is widely employed across multiple disciplines, such as medicine, education and computer science, for the purpose of assessing the readability of written materials and assuring their suitability for the target readers.^37^
4. SMOG: The algorithm determines the reading proficiency of a given text by analysing the frequency of polysyllabic words within a representative sample of the text. This formula yields a grade level output, which signifies the number of years of formal education necessary to comprehend a given paragraph.^38^
5. Flesch Kincald grade level (FKGL): The FKGL is a formula used to assess the readability of a text by analysing the average syllables each word and the average words each sentence. The system provides an output showing the grade level, which indicates the quantity of years of academic study necessary to comprehend a certain text.^39^, ^40^
6. Flesch reading ease (FRE): The scoring system is derived by computing the mean syllable count per word and the mean word count per sentence. The FRE score is a numerical scale that spans from 0 to 100, where higher scores are indicative of a higher level of reading. This metric is highly valuable in evaluating the simplicity of access and comprehensibility of text.^41^, ^42^

All of the tests, except the FRE test, give a score that reflects the approximate grade level of the text or written document required to understand it in the United States. For instance, a score of 6 corresponds to a reading level equivalent to the sixth grade. The Flesch Reading Ease uses a numerical score of 0 to 100. FRE score as like this: 0–30 is very difficult, 30–50 is difficult, 50–60 is fairly difficult, 60–70 is standard, 70–80 is fairly easy, 80–90 is easy and 90–100 is very easy.^43^

### 2.1 Statistical analysis

In this research, statistical analyzes were performed using Number Cruncher Statistical System software (version 2007; NCSS, Utah, USA).

Descriptive statistical methods were used to assess the distribution of each variable: mean, standard deviation, 95% CI, median, minimum–maximum values and Kolmogorov–Smirnov and Shapiro–Wilk tests for normality. These distributions were evaluated thus. Results were considered at a significance level of *p*‐value < .05.

The primary outcome of this study was to determine the readability of the home pages of the 10 most prominent patient‐oriented websites focused on TMD. This was measured using six different readability assessments to provide a comprehensive evaluation of the text's complexity and accessibility. The secondary outcomes included identifying the educational level necessary to comprehend the content on these websites and comparing the results across different readability assessments to ensure consistency and reliability of the findings.

## 3 RESULTS

Out of the 137 webpages identified during the initial search on Google Chrome and 38 out of 137 results were YouTube videos. We selected the top 10 patient‐oriented websites to ensure that the most accessible and widely read websites were evaluated taking into account the inclusion and exclusion criteria. This selection was based on the prominence of these websites in the search results and their focus on providing information to patients rather than healthcare professionals.

A thorough overview of the research findings, encompassing an analysis of the quantity of letters and words included within the texts in the websites, is presented in Table 1.

**TABLE 1:** Assessment of the comprehensibility of the top 10 websites on temporomandibular disorders.

Websites URLs	Number of characters	Number of words	Gunning fog index	Coleman Liau index	Flesch Kincald grade level	Automated readability index	Simple measure of gobbledygook	Average grade level	Flesch reading ease
https://www.hopkinsmedicine.org/health/conditions‐and‐diseases/temporomandibular‐disorder‐tmd	3348	671	13.8	12	11.6	12.6	10.8	12	49.3
https://www.mayoclinic.org/diseases‐conditions/tmj/symptoms‐causes/syc‐20350941	7318	1458	13.8	12	10.5	11.3	10.3	11	52.3
https://my.clevelandclinic.org/health/diseases/15066‐temporomandibular‐disorders‐tmd‐overview	7109	1387	10.1	12	7.3	7.7	7.6	8	60.7
https://www.nidcr.nih.gov/health‐info/tmd	11 276	2227	12.5	12	9.8	10.1	9.5	10	52.2
https://www.webmd.com/oral‐health/temporomandibular‐disorders‐tmd	6769	1496	9.1	9	6.8	7.3	6.7	8	72.8
https://www.healthline.com/health/tmj‐headache#outlook	3887	786	12.2	11	10	11	9	11	55.9
https://www.pennmedicine.org/updates/blogs/health‐and‐wellness/2020/september/tmj‐pain‐relief‐8‐best‐practices‐to‐help‐manage‐tmd	3844	787	14.7	11	12.9	15.1	10.2	13	51
https://www.physio‐pedia.com/Temporomandibular_Disorders	13 714	2701	14.6	12	11.7	11.4	11.1	12	43.3
https://www.aafp.org/pubs/afp/issues/2015/0315/p378.html	12 498	2267	17.4	15	14.2	12.3	12.2	13	20.3
https://en.wikipedia.org/wiki/Temporomandibular_joint_dysfunction	16 031	2981	16.7	14	14.3	14.2	12.8	14	28.9

Kolmogorov–Smirnov and Shapiro–Wilk tests were conducted to verify the normal distribution of variables for all the measures (*p* > .05) (Table 2).

**TABLE 2:** Evaluation of the distribution of variables in utilised tests.

Tests of normality	Kolmogorov–Smirnov test			Shapiro–Wilk test		
	Statistic	df	*p*	Statistic	df	*p*
Number of characters	0.210	10	.200	0.912	10	.294
Number of words	0.186	10	.200	0.917	10	.333
Gunning fog index (GFI)	0.147	10	.200	0.965	10	.838
Coleman Liau index (CLI)	0.173	10	.110	0.898	10	.211
Flesch Kincald grade level (FKGL)	0.133	10	.200	0.942	10	.580
Automated readability index (ARI)	0.152	10	.200	0.954	10	.719
Simple measure of gobbledygook (SMOG)	0.138	10	.200	0.972	10	.913
Average grade level	0.161	10	.200	0.924	10	.388
Flesch reading ease (FRE)	0.217	10	.200	0.938	10	.534

Abbreviation: df, degrees of freedom.

Table 3 presents the mean values of various readability indices, including the CLI, FKGL, ARI, and Simple Measure of Gobbledygook. These outcomes are indicative of the grade level in the United States that is required for comprehending the text. The mean value for the CLI is 12.00 ± 1.63, for the FKGL is 10.91 ± 2.57, for the ARI is 11.30 ± 2.50, and for the SMOG is 10.02 ± 1.90.

**TABLE 3:** Descriptive statistical values of the indexes used in the tests.

	Mean ± SD	95% ICC	Median	Min‐Max
Number of characters	8579.40 ± 4513.66	(5350.52–11808.28)	7213.50	(3348–16 031)
Number of words	1676.10 ± 827.47	(1084.16–2268.04)	1477	(671–2981)
Gunning fog index (GFI)	13.49 ± 2.62	(11.61–15.37)	13.80	(9.10–17.40)
Coleman Liau index (CLI)	12.00 ± 1.63	(10.83–13.17)	12	(9–15)
Flesch Kincald grade level (FKGL)	10.91 ± 2.57	(9.07–12.75)	11.05	(6.80–14.30)
Automated readability index (ARI)	11.30 ± 2.50	(9.52–13.09)	11.35	(7.30–15.10)
Simple measure of gobbledygook (SMOG)	10.02 ± 1.90	(8.66–11.38)	10.25	(6.70–12.80)
Average grade level	11.20 ± 2.04	(9.74–12.66)	11.50	(8–14)
Flesch reading ease (FRE)	48.67 ± 15.04	(37.91–59.43)	51.60	(20.30–72.80)

Abbreviations: ICC, intraclass correlation coefficient; SD, standard deviation.

The average grade level for each website is determined by calculating the average of four readability indexes: CLI, FKGL, ARI and SMOG. The findings indicate that the websites related to ‘temporomandibular disorders’ that are accessed most frequently necessitate ‘average grade level’ of 11.20, accompanied by a standard deviation of 2.04, in order to be comprehended. Similarly, these websites require an average of 13.49 years of formal education (GFI), with a standard deviation of 2.62, for ease of understanding (Table 3).

The FRE index is used to ascertain the reading ease of the text. The Mean index score stood at 48.67, with a standard deviation 15.04. According to FRE, index of the most difficult content was 20.3. *Wikipedia* was second most difficult to read, with a score of 28.9. This indicates an approximate reading age of 17–21 years (Table 1). These results suggest that the comprehension of the websites assessed presented a significant challenge (Table 3).

It is simply necessary to note that all health‐related material has to be understandable and within reach of everybody, with a threshold requirement being that expected of a ninth‐grade graduate—high school. Based on the available data, it is evident that only two websites provide this particular feature (Average Grade Level <9) (Table 1).

## 4 DISCUSSION

Individuals often choose the practice of visiting many websites in order to enhance their comprehension of specific topics and to verify the accuracy and reliability of the material they understand. The present study brings attention to the disparity that exists between the level of readability found in website content and the specific informational requirements of individuals suffering from pain. Despite the widespread availability of patient‐oriented websites, it is inadequate in effectively conveying a comprehensive understanding of patients' medical conditions, their relevance, and the potential consequences for their overall health.^44^ Hence, it is essential to ensure that accessible information from various websites can be made available to foster better decision‐making among a more significant percentage of the populace.^45^

The findings of the conducted study indicate that the homepage texts of the top 10 websites associated with TMD exhibited a level of readability and comprehensibility that necessitated a minimum degree of education at the university level. This finding demonstrates that the websites did not hit the recommended readability index set by the USA National Institutes of Health for a website to be written at the understanding level of a person who has completed 6–7 years of education. It is suggested that health information be pitched at an average of 11 years of age, which equates to a sixth‐grade level, as recommended by the NHS Health Education England and the Office for National Statistics. A recommended range for the FRE score was established between 80 and 90.^46^ However, the results of the study suggest that the quantity of internet resources associated with TMD exceeds the recommended 6th‐grade reading level and FRE score.

The study identified *Wikipedia* as one of the websites that had low readability, consistent with previous research.^47^ Wikipedia has been recognised as an important and frequently used source of health information, frequently appearing on the initial page of search results.^48^ This suggests that individuals seeking health information may encounter complex and challenging content at the initial stages of their information search.^9^, ^10^, ^11^, ^49^

It also implies that TMD's therapeutic approach can differ from one patient to another. The orthodontic professional views the assessment of a most opportune case considering the patient's particular requirements for treatment. Hence, it is advisable that individuals experiencing symptoms of TMD collaborate with a specialist to develop an individualised treatment strategy. Health information providers must to integrate readability analysis into their content creation process of websites, by integrating clear phrases and use more accessible vocabulary so that accurate diagnosis, and appropriate treatment can be ensured.^50^, ^51^, ^52^, ^53^

Given the findings that most websites on TMD are not easy to read and understand, we recommend several strategies for designing more accessible websites. Firstly, the text and figures should be tested with different groups, including patients with TMD, healthy individuals, and general practitioners. This user testing can provide valuable feedback on the clarity and comprehensibility of the content in the website.

Additionally, collaborating with a professional web designer can enhance the format and presentation of the information, making it more accessible and enticing. Web designers can help optimise the layout, use of colours, and interactive elements to improve user engagement and understanding. It would be beneficial to investigate whether the websites were created by professional web designers. Websites designed by professionals are more likely to follow best practices in usability and accessibility, which can significantly impact the effectiveness of the health information provided. However, we did not check whether the websites were created by professional web designers. Further research can explore this aspect to determine its impact on readability and user experience.

### 4.1 Limitations

There exist certain limitations that are largely inevitable and must be taken into account while interpreting these findings. Initially, the findings were derived from a cross‐sectional examination conducted at a particular moment in time. It is widely recognised that websites undergo regular updates, and search engines dynamically adjust their algorithms based on website traffic patterns and the past search activities of particular users. Moreover, despite the fact that the conducted searches did not pertain to a time‐sensitive topic, a recommendation for future investigations on readability analyses would be to perform the search at various time intervals in order to ascertain if the outcomes exhibit any general changes. The findings, though, indicate the accessibility of health‐related information offered by prominent organisations that experience substantial internet visitation. Another constraint is associated with the choice of search phrases utilised. The search term employed by researchers was ‘temporomandibular disorders’; however, it is recognised that certain individuals making internet searches may utilise words or particular symptoms. Medical terminology is considered unavoidable in pages that show treatment options for a medical condition. It indicates that there might be websites that exhibit higher readability scores as a result of the more number of syllables in the words used. However, the authors used different readability analysis techniques that combined several factors, such as sentence length, word length, and syllables, to come up with a score for the calculation of the average value.

We have only tested readability tests. We did not consider the coherence and comprehensibility of the text on this website. These are potential areas of inquiry that require further investigation if a comprehensive understanding of readers' perceptions and comprehension of the content is to be attained. However, the results of our study suggest that the general population may have difficulties in comprehending health‐related material accessible in the website, which can result in misconceptions, delayed identification of medical conditions, and ultimately, inferior health results.

## 5 CONCLUSIONS

As emphasised in the research, the accessibility of medical information in the websites might be a considerable obstacle for individuals especially those with little proficiency in medical terminology. Hence, it is essential for orthodontists to offer guidance and assistance to their patients regarding the acquisition of medical information from websites.

Orthodontists can also inform patients about medical terms and guide them to obtain information from reliable sources such as university hospitals, official health organisations, health associations and well‐known health websites via explaining medical terminology to the patients.

The websites serve as a valuable repository of knowledge in various domains, including health‐related matters. However, it is imperative for individuals to exercise attentiveness when accessing such information. When seeking accurate and comprehensive information regarding medical conditions such as TMD, it is advisable to consult orthodontists or healthcare professionals, as their expertise and knowledge render their insights more dependable and easily comprehensible. This measure will effectively reduce the risk of self‐inflicted injury to the individual.

## AUTHOR CONTRIBUTIONS

Giuseppe Minervini designed the research study. Hande Uzunçıbuk performed the research. The data were analysed by Vincenzo Ronsivalle and Marco Cicciù. Maria Maddalena Marrapodi and Hande Uzunçıbuk wrote the manuscript. All authors contributed to the editorial changes in the manuscript. All authors read and approved the final manuscript.

## CONFLICT OF INTEREST STATEMENT

The authors declare no conflict of interest.

## Data Availability

The manuscript contains a comprehensive presentation of all the data. The datasets that were analysed can be obtained from the respective author upon a reasonable request.
